# Dendrimeric Template of *Plasmodium falciparum* Histidine Rich Protein II Repeat Motifs Bearing Asp→Asn Mutation Exhibits Heme Binding and β-Hematin Formation

**DOI:** 10.1371/journal.pone.0112087

**Published:** 2014-11-14

**Authors:** Pinky Kumari, Dinkar Sahal, Virander S. Chauhan

**Affiliations:** Malaria Research Laboratory, International Centre for Genetic Engineering and Biotechnology, Aruna Asaf Ali Marg, New Delhi, 110067, India; RWTH Aachen University, Germany

## Abstract

*Plasmodium falciparum* (*Pf*) employs a crucial *Pf*HRPII catalyzed reaction that converts toxic heme into hemozoin. Understanding heme polymerization mechanism is the first step for rational design of new drugs, targeting this pathway. Heme binding and hemozoin formation have been ascribed to *Pf*HRPII aspartate carboxylate-heme metal ionic interactions. To investigate, if this ionic interaction is indeed pivotal, we examined the comparative heme binding and β-hematin forming abilities of a wild type dendrimeric peptide BNT1 {harboring the native sequence motif of *Pf*HRPII (AHHAHHAADA)} versus a mutant dendrimeric peptide BNTM {in which ionic Aspartate residues have been replaced by the neutral Asparaginyl residues (AHHAHHAANA)}. UV and IR data reported here reveal that at pH 5, both BNT1 and BNTM exhibit comparable heme binding as well as β-hematin forming abilities, thus questioning the role of *Pf*HRPII aspartate carboxylate-heme metal ionic interactions in heme binding and β-hematin formation. Based on our data and information in the literature we suggest the possible role of weak dispersive interactions like N-H···π and lone-pair···π in heme binding and hemozoin formation.

## Introduction

Malaria is one of the most severe public health problems worldwide. In 2010, malaria caused an estimated 216 million clinical episodes, and 655,000 deaths [1]. Children under five and pregnant women are the most affected groups. Five species of *Plasmodium* have been recognized to infect humans; of whom *Plasmodium falciparum* (*Pf*) is the most virulent and most recently evolved [2]. Antimalarial drug resistance and lack of new antimalarials are major challenges to malaria control.

Toxic heme constitutes the inevitable outcome of the penchant of the malaria parasite to digest hemoglobin for its nutritional requirements in the blood stage of its life cycle. This process which occurs in the miniscule volume of a few femtoliters in the food vacuole of the parasite leads to a massive accumulation of heme leading to concentration as high as 300–500 mM [3]. The highly successful proliferative life cycle of the malaria parasite notwithstanding such a toxic mileu, owes a lot to the efficient heme detoxification strategies evolved by the parasite. One such strategy involves the deployment of “heme mopper proteins” such as the histidine rich proteins (HRP) II and III [4], which are characterized by the occurrence of repetitive histidine rich heme binding sequence motifs; that occur throughout the sequence of these natively unfolded proteins. Hemozoin is apparently identical to β-hematin (an *in vitro* synthesized complex of heme), which is a reciprocal dimer of Fe(III)PPIX in which propionate groups of each heme coordinates with the Fe(III) centre of the neighboring heme and the resulting dimers are linked through hydrogen bonds across the second propionic acid groups [5]. Malariologists and drug developers have been fascinated by this pathway of heme detoxification since it is absent in humans. Currently two hypotheses concerning hemozoin formation viz via proteins like the Histidine rich protein (HRP) and the heme detoxification protein (HDP) [6] or via the hydrophobic mileu provided by lipids are in vogue [7]. However the exact mechanism of hemozoin formation in *P.falciparum* still remains unclear [8] and in particular the role of HRPs in hemozoin formation is still unknown and open to investigation.

**Figure 1:**
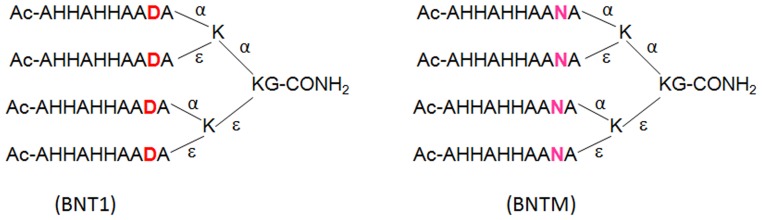
Sequence of dendrimeric peptides used in this study. All aspartic acid (D) residues of BNT1 have been substituted by asparagines (N) in BNTM. α and ε denote amino groups of lysine.

*Pf*HRPII is a 30 kDa protein, 85% of which consists of repeat sequences like Ala-His-His-Ala-His-His-Ala-Ala-Asp-Ala [9], [10]. *Pf*HRPII has been shown to bind Fe(III)PPIX with significant changes in its conformation [11], [12]. While synthetic linear peptides corresponding to these sequence motifs show heme binding but no hemozoin formation, K-K2 dendrimers called bionucleating templates (BNTs) exhibiting four branches of this linear motif have been shown to not only bind heme but also catalyze β-hematin formation [13]. At food vacuole pH (4.5–5.5) *Pf*HRPII-heme binding has been assumed to be via aspartate carboxylate-metal ionic/coordinate interactions and hemozoin formation has been correlated with aspartate binding [12]. In the present work, we wanted to study if aspartate carboxylate-heme metal ionic interactions are indeed crucial to heme binding and β-hematin formation. Towards this, we have replaced the four aspartyl residues of BNT1 by asparginyl residues to obtain BNTM (Figure 1). Here we report that aspartate metal ionic interactions are not essential since both BNT1 and BNTM exhibit comparable heme binding as well as β-hematin forming abilities.

## Materials and Methods

### Materials and Sample Preparation

All solvents and reagents were used as received unless specified. Fmoc (Fluorenylmethoxycarbonyl) amino acid derivatives {Fmoc-Ala-OH, Fmoc-Asp (otBu), Fmoc-Asn (trt), Fmoc-His (trt)}, Rink Amide methylbenzhydrylamine {(100–200 mesh), (MBHA)} resin and O-Benzotriazole-N, N, N′, N′-tetramethyl-uronium-hexafluoro-phosphate (HBTU) for peptide synthesis were from Nova Biochem. Fmoc-Glycine and Fmoc-L-Lys-(Fmoc)-OH were from Chem-Impex International and Orpegen Pharma respectively. Dimethylformamide (DMF), Piperidine, N,N'-Diisopropylcarbodiimide (DIPCDI), 1-Hydroxy Benzotriazole Monohydrate (HOBt), N,N-Diisopropylethylamine (DIEA), Trifluoroacetic acid (TFA), Triisopropylsilane (TIS), Dimethyl sulfoxide (DMSO), Heme, HEPES and Polyhistidine were from Sigma. Acetonitrile (ACN) was from J. T. Baker. General reagents used were of analytical grade.

Fresh stock solution of heme was made by dissolving recrystallized heme in DMSO and the solution was filtered through a 0.45 µm syringe filter. Concentration of the resulting solution was determined spectroscopically in DMSO using an extinction coefficient of 170 mM^−1^cm^−1^ at 404 nm [14]. BNT1 and BNTM stock solutions were made in 20 mM sodium acetate buffer (pH 5), and stored at −20°C.

### Synthesis of Bionucleating Peptide Dendrimer Templates

K-K2 peptide dendrimer templates (BNT1 and BNTM) were synthesized as C-terminal amides using standard Fmoc chemistry on Rink amide MBHA resin in manual mode using Fmoc amino acid/HBTU/HOBt/DIEA in the molar ratio of 1∶1∶1∶2 in DMF. Fmoc-Lys (Fmoc)-OH was used to create a branch core for the synthesis of the K-K2 peptide dendrimers. Both Fmoc groups of Fmoc-Lys (Fmoc)-OH-resin complex were removed by treatment with 20% piperidine in DMF giving rise to two (α and ε) amino group. The synthesis of peptide dendrimers was accomplished on a K-K2 core generated by coupling of Fmoc-Lys (Fmoc)-OH to the two free amino groups on the lysine coupled resin synthesized as described above. Four free amino groups (α and ε, for each lysine derivative) so obtained, were used for simultaneous synthesis of four identical chains of the same peptide as shown in Figure 1. Couplings of Fmoc amino acids were done in DMF at 4-fold molar excess of activated amino acids. Both coupling of Fmoc derived amino acids and Fmoc deprotections were monitored by Kaiser Test [15]. Amino termini were acetylated using acetic anhydride/pyridine/DMF (1∶1∶5 v/v) for 20 minutes, after the completion of assembly of the peptides. After acetylation, the resin was washed extensively with DMF, DCM and methanol and dried in a desiccator under vacuum. Peptides were cleaved from resin by stirring the resin in a cleavage mixture (95% TFA, 2.5% water and 2.5% TIS) for 2 hrs at room temperature. The suspension was filtered using a sinter funnel and resin was washed with the cleavage mixture. Rotary evaporator was used to evaporate TFA and the peptides were precipitated with chilled ether. The precipitated peptides were harvested by filtration on a sinter funnel, dissolved in a minimum volume of 20% CH_3_CN and lyophilized.

### Peptide Purification and Mass Spectrometry

Crude K-K2 peptide dendrimers were purified on a preparative reversed phase C_18_ column (Shimadzu, PRC-ODS column, 150×20 mm, 15 µ) using 0.1% TFA/water (solvent A) and 0.1% TFA/acetonitrile (solvent B). Linear gradient 5–60% B (Flow rate 6 mL/min over 27.5 minutes) was used for purification of the two peptide dendrimers. Analytical purity of purified BNT1 and BNTM was assessed by chromatography on a reversed phase C_18_ column (Microsorb, 15×4.6 mm, 15 µ) using 0.1% TFA/water (solvent A) and 0.1% TFA/acetonitrile (solvent B) and running a 5–45% linear gradient over 20 minutes at a flow rate of 1 mL/min. Dual Wavelength detector was set at 214/280 nm. The identities of peptide dendrimers were confirmed by electrospray ionization mass spectrometry (ESI-MS) at ICGEB, New Delhi. Experimental conditions used during ESI-MS of BNT1 and BNTM were solvent: 50% acetonitrile/0.1% formic acid, positive ionization mode, voltage settings (4 kilowatt) and instrument Orbitrap velos pro (Thermo Fisher Scientific).

### Stability of BNTM

BNTM (100 µM) in 100 µL of buffer (500 mM sodium acetate, pH 5) was incubated under β-hematin forming conditions (260 rpm, 24 hrs, and 37°C). The treated sample of BNTM {BNTM (t)} was diluted to 1 mL with 0.5% acetic acid and passed (2 times) through C_18_ mini spin column {(Pierce), (equilibrated with 0.5% acetic acid)}. Column was washed with 0.5% acetic acid and elution of BNTM (t) was done with 80% acetonitrile solution. After evaporation and lyophilization, solid BNTM (t) was analyzed by ESI-MS.

### Titrimetric determination of heme binding sites

Difference spectroscopy (heme titrations) studies were done as described earlier [16]. Spectra were recorded on a Perkin-Elmer Lambda Bio20 spectrophotometer between 250–700 nm at a speed of 120 nm/min and a slit width of 1 nm. Heme was titrated in two separate experiments, for binding to BNT1 and BNTM. A hemin solution (2 mM in DMSO) was simultaneously titrated into (i) a solution of 5 µM of BNT1/BNTM in 3 mL of 200 mM HEPES, pH 7 and (ii) a reference cuvette containing HEPES buffer only. Heme was titrated in 2 µM increments. After each addition of heme, the samples in the two cuvettes were mixed and allowed to stand for 5 minutes to allow complete binding before recording of difference spectra. Difference absorption spectra were recorded after each incremental addition of heme. Concentration of DMSO was kept below 2% in all reaction mixtures. The difference spectra had their maxima and minima at 415 nm and 360 nm respectively. Heme-binding curves of BNT1 and BNTM were generated from the difference absorption spectra by plotting A_415_ vs the moles of heme/mole of peptide template. Horizontal dotted lines were drawn to connect the plateau points of each graph to the Y axis. At the point of intersection, vertical arrows were drawn to obtain the values of heme/peptide.

### BNT1 vs BNTM: Comparative kinetics of heme binding

Spectra were recorded on a Perkin-Elmer Lambda Bio20 spectrophotometer between 250–700 nm at a speed of 120 nm/min and a slit width of 1 nm. Heme was titrated in two separate experiments, for binding to BNT1 and BNTM. Heme solution (in DMSO) was simultaneously titrated into (i) a solution of 5 µM of BNT1/BNTM in 1 mL of 200 mM HEPES, pH 7 and (ii) a reference cuvette containing HEPES buffer only. Heme was titrated in 4 µM increments. Difference absorption spectra were recorded at different time points (0, 0.5, 1, 2, 3, 4, 5 minutes), after each incremental addition of heme. Concentration of DMSO was kept below 2% (v/v) in all reaction mixtures. Graphs of comparative kinetics of heme binding to BNT1/BNTM were made in excel by plotting A_415_ vs time.

### BNT1 vs BNTM: Comparative kinetics of Hemozoin Formation

A solution of heme (45 mM) in DMSO was aliquoted to a final concentration of approximately 300 µM into microfuge tubes. Solutions of BNT1 and BNTM in acetate buffer (20 mM, pH 5) were aliquoted into their respective tubes to a final concentration 5 µM in a final volume of 1.5 mL made with 500 mM sodium acetate buffer (pH 5). Heme alone taken under identical conditions acted as the negative control. The final DMSO concentration in all reactions was kept below 2%, which did not interfere with hemozoin formation. Each assay was set up in triplicates and incubated for different time points (0, 12, 18, 24 and 48 hours) on a rotating shaker (260 rpm) at 37°C. After the completion of each time point, the reaction was stopped by adding 10 µL of 10% SDS in phosphate-buffered saline (PBS). Reaction mixture was vortexed for 5 minutes and centrifuged (13000 rpm, 15.7 rcf, 60 min, and 25°C). Pellets so obtained were suspended in 1.5 mL of 2.5% SDS in (PBS) and incubated for 1 hr with shaking (260 rpm) at 37°C followed by centrifugation as above. Pellets were resuspended in 1.5 mL of 100 mM sodium bicarbonate (pH 9.2) by vortexing. The resulting mixture was centrifuged as above and supernatant was aspirated out. Two additional bicarbonate washes were given as above. Bicarbonate washes are known to remove free heme and non hemozoin oligomers adsorbed to hemozoin [17]. Pellets were then washed with distilled water (2 washes) and finally with 95% ethanol. For the estimation of β-hematin, pellets were solubilized in 0.1 N NaOH/2.5% SDS (2 mL) and absorbances were measured at 401 nm. Excel graphs depicting absorbance 401 nm versus time were plotted and data were evaluated by standard deviation and t test.

### Spectroscopic Characterization of Hemozoin

Fresh stock solution of heme was made by wetting recrystalized heme (Sigma) in 1N NaOH, diluting with 100 mM HEPES (pH 7). Resulting solution was vortexed, centrifuged (13000 rpm, 15 min, 25°C), supernatant filtered through 0.22 µm syringe filter and concentration of filtrate was determined spectroscopically in 100 mM HEPES (pH 7) using molar extinction coefficient at 400 nm of 62 mM^−1^cm^−1^. Heme (final concentration 50 µM) was aliquoted into eppendorf tubes. BNT1 and BNTM (Peptide dendrimers, 20 nmoles) were added and finally the volume was brought to 1.5 mL with 500 mM sodium acetate buffer (pH 5). Heme alone (negative control) was also run simultaneously. Each assay was set up in quadruplicate and incubated at 37°C for 36 hours on a rotating shaker (260 rpm). The reaction was stopped by adding 10 µL of 10% SDS in PBS. Reaction mixture was vortexed for 5 minutes and centrifuged (13000 rpm, 60 min, 25°C). Pellet was resuspended in 1.5 mL of 100 mM sodium bicarbonate (pH 9.2) in 2.5% SDS by vortexing and sonication. The resulting solution was centrifuged (13000 rpm, 15.7 rcf, 60 min, 25°C). Two additional bicarbonate washes were given as above. Pellets were washed twice with 2.5% SDS by resuspending, vortexing, sonication, and centrifugation (13000, 15.7 rcf, rpm, 60 min, and 25°C). In order to characterize hemozoin by spectroscopy, pellets were resuspended in 500 µL of 2.5% SDS, vortexed for 5 min and incubated overnight. Next day spectra were recorded between 300 nm–700 nm. Hemozoin suspensions were then made 0.02 N with NaOH to obtain typical hematin UV-visible spectrum in each case [18]. β- hematin (an *in vitro* synthesized complex of heme and apparently identical to hemozoin [19]) spectrum was also recorded under similar condition and used as reference.

### FT-IR Spectroscopy

To obtain IR spectra, KBr pellets were prepared from dried samples of BNT1,BNTM, polyhistidine mediated heme aggregates and β-hematin spectra were acquired for 100 cycles with a Fourier-transform IR spectrometer (Perkin-Elmer).

## Results and Discussion

### Characterization of K-K2 Peptide Dendrimers (BNT1, BNTM) by RPHPLC and Mass Spectroscopy

BNT1 and BNTM were purified by RPHPLC using acetonitrile-water gradient. The observed retention times (min) was 18 and 17 for BNT1 and BNTM respectively (Figure S1). The identities of these two BNTs were confirmed by mass spectrometry. Observed mass values were 4703 Da for BNT1 (Expected mass- 4704 Da) and 4698 Da for BNTM (Expected mass- 4700 Da) {Figure S2, panels A and B}.

### Stability of BNTM

There is possibility of deamidation of asparagine moieties of BNTM to aspartate under conditions used to promote hemozoin formation. This could lead to in situ generation of BNT1 from BNTM. So stability of BNTM treated {BNTM (t)} under high ionic strength and acidic condition (500 mM sodium acetate, pH 5) was analyzed by mass spectrometry. The observed mass values for BNTM and BNTM (t) were 4698 Da and 4699 Da respectively {Figure S2, panels B and C}. This observed difference in mass of 1 Dalton between BNTM and BNTM (t) is most likely to be due to low MS resolution and not due to deamidation since selective hydrolysis of one out of four asparaginyl residue of BNTM is quite unlikely. The nearly identical masses of BNTM and BNTM (t) confirm the stability of BNTM under acidic and high ionic strength conditions used to promote hemozoin formation.

### BNT1 and BNTM: Spectroscopic titrimetric determination of heme binding sites and comparative kinetics of heme binding

**Figure 2:**
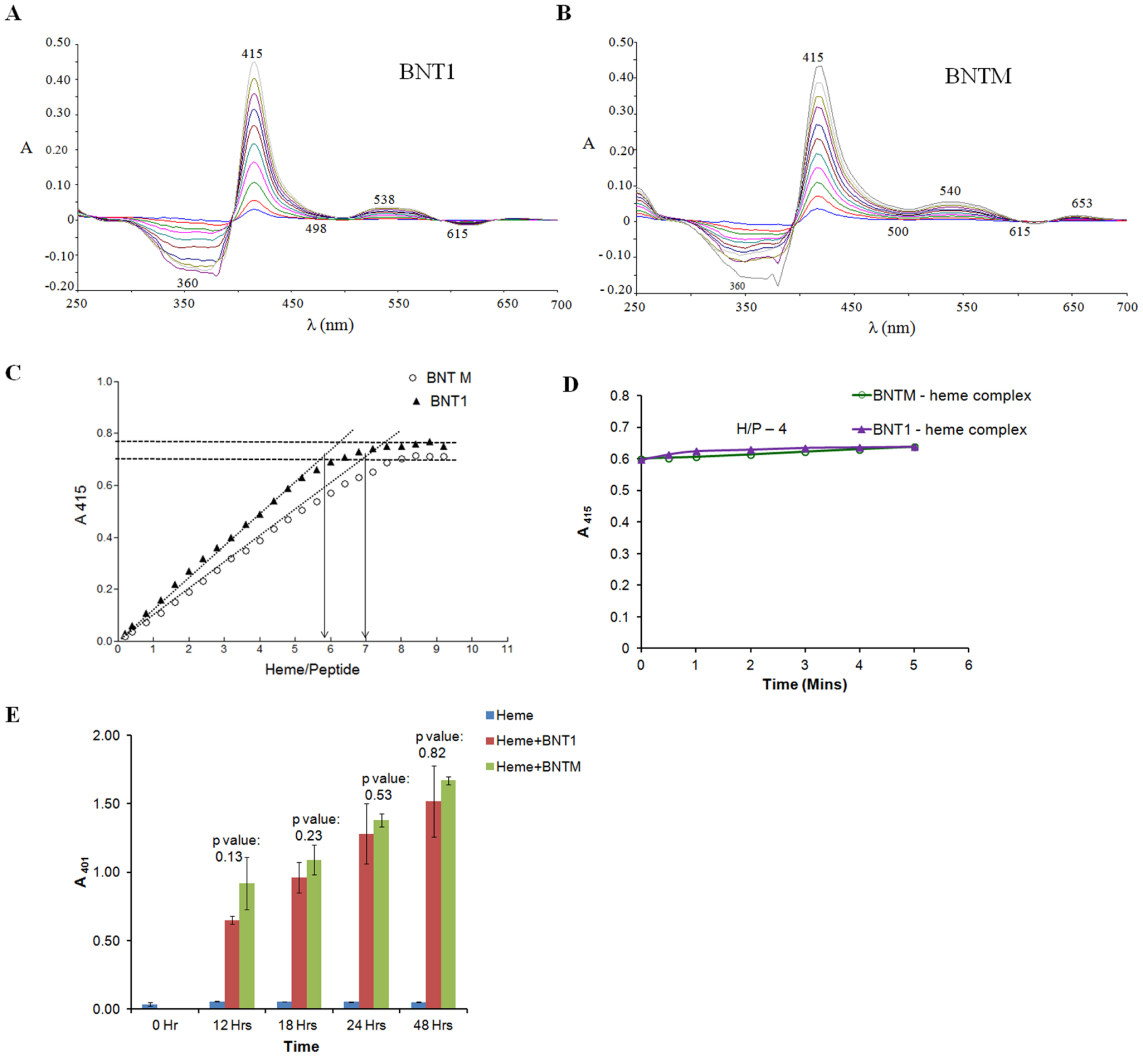
Difference Spectroscopic Titration and formation of β-hematin. Panels A and B show difference absorption spectra of heme binding to BNT1 and BNTM respectively. Panel C shows heme-binding curves of BNT1 and BNTM generated from the difference absorption spectra by plotting A_415_ vs the moles of heme/mole of peptide template. Horizontal lines were drawn connecting the Y axis to the plateau region of the binding curves. At the point of intersection, vertical arrows were drawn to obtain the values of heme/peptide.Panel D shows comparative kinetics of heme binding to BNT1/BNTM (at heme: peptide molar ratio of 4) studied by difference spectroscopy. Data shows intensity of Soret peak as a function of time. Panel E shows comparative kinetics of β-hematin formation in presence of BNT1/BNTM.

To examine if the synthetic peptide dendrimers bind with heme, we analyzed changes in the absorption spectrum of heme (λ_max_ 384 nm) after mixing it with BNT1 or BNTM. The difference spectra (Figure 2, Panels A and B) showed the principal Soret peak at 415 nm for BNT1-heme and BNTM-heme complexes. The heme-binding sites on BNT1 and BNTM were titrated with increasing amounts of heme and difference absorption spectra were recorded at varying heme concentrations. Red shift (λ_max_ 384 to 415 nm) was observed upon heme binding to BNT1/BNTM. The absorbance at 415 nm increased until it reached a plateau. Analysis of heme binding curves indicated binding stoichiometries of 5.8±0.6 and 6.75±0.4 for BNT1/BNTM: heme respectively {Figure 2, panel C}. The BNT1-heme stoichiometry observed here is consistent with a previous study of BNT1-heme binding [13].

No significant difference was found in heme binding stoichiometries of BNT1/BNTM binding at 415 nm. Absorbance at 415 nm corresponds to the Soret peak of the stable bis-histidyl heme complex and does not provide direct information on Asp/Asn contributions to heme binding. To study the contribution of Asp/Asn in kinetics of bis-histidyl complex formation, comparative kinetics of heme binding potential of BNT1 vs BNTM was studied. As shown in (Figure 2, panel D) there is no significant difference in the kinetics of heme binding by these two peptides.

### BNT1 vs BNTM: Comparative kinetics of hemozoin formation

Heme binding to a template protein is essential for formation of a supramolecular structure like hemozoin. However heme binding alone is not sufficient for β-hematin/hemozoin formation. For instance, polyhistidine binds multiple monomers of heme but fails to form β-hematin [4]. Changes in secondary structure of template protein like *Pf*HRPII have been observed upon *Pf*HRPII-heme binding [12], [20], suggesting that favorable secondary structures on the protein template may be essential to promote hemozoin formation. Acidic pH (4.5–5.5) also seems to play a crucial role in hemozoin formation as half of the propionate side chains of heme are deprotonated at this pH, thus allowing the formation of iron carboxylate metalloester bonds between propionate side chains of one heme with the central Fe^3+^of another heme [19], [5].

**Figure 3:**
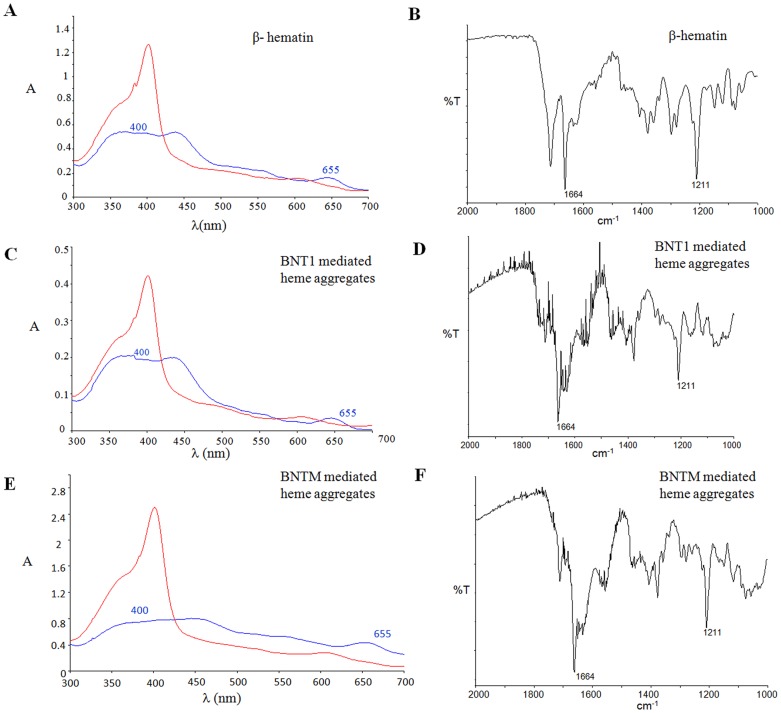
Spectroscopic characterization of bicarbonate stable heme aggregates formed in presence of BNT1/BNTM. Panels A, C and E show UV-visible spectra of β-hematin and BNT1/BNTM mediated bicarbonate stable heme aggregates respectively. These aggregates were incubated overnight in 2.5% aqueous SDS prior to recording spectra (blue line). Note the broad Soret (400 nm) and the weak but characteristic peak at 655 nm. Typical hematin spectra (sharp Soret at 400 nm - red line) were obtained when each suspension was made 0.02 N with respect to NaOH. Panels B, D and F show the FTIR spectra of β-hematin and BNT1/BNTM mediated bicarbonate stable heme aggregates respectively. Peaks at 1664 cm^−1^ and 1210 cm^−1^ in FTIR spectrum of heme aggregates mediated by BNT1 and BNTM confirm their identity as β-hematin.

Difference spectroscopy of heme binding to BNT1 and BNTM motivated us to investigate their β-hematin forming abilities. Both BNT1 and BNTM led to *in vitro* β-hematin formation when incubated with heme at pH 5 (Figures 2 and 3). Yield of β-hematin increased with increasing incubation time in case of both BNT1 and BNTM. Statistical analysis (p value) at each time point confirmed that there is no significant difference in the level of β-hematin formed by BNT1 vs BNTM (Figure 2, panel E) suggesting that aspartate (BNT1) and asparagine residues (BNTM) do not differentially impact hemozoin formation.

UV-Visible spectroscopy was done to examine if these bicarbonate stable BNT1/BNTM-heme aggregates have features of β-hematin [18]. Heme aggregates formed by BNT1/BNTM (Figure 3, Panels C and E) upon overnight incubation in 2.5% aqueous SDS, exhibited an absorption maximum at 655 nm and a broad Soret band centered at ∼400 nm. When NaOH was added to these suspensions, the resulting solutions exhibited sharp Soret band centered at 400 nm typical of hematin. The two absorbances (655 and 400 nm) have been considered as spectroscopic signatures associated with β-hematin [18] and their presence in panels C and E therefore suggests that the aggregates formed in presence of BNT1/BNTM are β-hematin. Peaks at 1664 cm^−1^ and 1210 cm^−1^ in FTIR spectrum [19] of heme aggregates mediated by BNT1 and BNTM further confirm their identity as β-hematin (Figure 3, Panels D and F).

The exact role of Aspartyl residues in *Pf*HRPII towards heme binding and hemozoin formation is unclear. Lynn et al [12] speculated that at pH∼5, aspartates of *Pf*HRPII may form ionic bonds with iron (Fe^3+^) of heme. However, our observations reported in the present work show that BNTM in which Aspartyl residues have been replaced by Asparginyl residues still binds heme and forms β-hematin (Figures 2 and 3). This suggests that iron-aspartate carboxylate ionic interactions may not be crucial for hemozoin/β-hematin formation. It is important to note that polyhistidine which binds multiple monomers of heme also gave rise to bicarbonate stable aggregates of heme. However these aggregates gave an IR spectrum which lacked the characteristics peaks (1210 cm^−1^ and 1664 cm^−1^) associated with β-hematin (Figure S3). This indicates that Aspartyl or Asparginyl residues may play a crucial role in catalyzing formation of hemozoin from the heme monomers anchored on a template that is rich in histidyl residues. This observation finds some support from the fact that while *Pf*HRPII is rich in Aspartyl residues {Aspartic acid (10.4%) and Asparagine (3.2%)} the *Pf*HRPIII is rich in Asparginyl residues {Aspartic acid (9.1%) and Asparagine (11.7%)}. Since both *Pf*HRPII and *Pf*HRPIII exhibit the ability to bind heme and initiate hemozoin formation [4], our results suggest that the Asparagine bearing motifs in *Pf*HRPIII may also be contributing to not only heme binding but also to hemozoin formation. This hypothesis is supported by the fact that in a comparative study of β-hematin formation, Lisa Pasierb [21] has reported that while the dendrimeric peptide BNTII (harboring 2 repeat units of 9 mer sequence Ala-His-His-Ala-His-His-Ala-Ala-Asp) was able to bind heme and promote β-hematin formation, the mutant peptide BNTIID (all Asp residues of BNTII replaced by Ala) lost both heme binding and β-hematin forming ability. This suggests that the lone pair of electrons present in the carbonyl oxygen of both Asp and Asn may be interacting with the heme π-electron cloud to facilitate heme binding followed by β-hematin formation.

Based on diverse evidences in literature, we propose that weak forces like N-H···π {NH of Asn side chain amide and π cloud (22π electrons) of heme and lone pair (lp)···π (lp from carbonyl of Asn/Asp and π cloud of heme) may assist in β-hematin formation by inducing specific structural changes required to initiate β-hematin formation. A survey of instances of lp···π interactions suggests that lone pairs can be contributed by a number of molecules like water [22], [23] ether [24] carbonyl moieties [25] or even anions [26], [27]. lp···π interactions has been found to stabilize Z-DNA structure [24]. This interaction has also been observed between water molecule and a cytosine base in RNA pseudoknot [23]. We hypothesize that just as in amide-benzene interactions where N-H···π is the dominant interaction rather than C = O···π interactions [28], similarly in BNTM-heme case, N-H···π may be the dominant interaction. Such N-H···π interactions (stabilization energy ∼3 Kcal/mol) [29] have been observed in studies of bovine pancreatic trypsin inhibitor (BPTI) [30], hemoglobin drug interactions [29], [31], and pyrroles [32]. Besides bringing heme molecules closer, these weak dispersive intermolecular interactions may also play a role in stabilizing heme-template complex and inducing specific structural changes in the template required to initiate hemozoin formation.

## Conclusions

Replacement of anionic Aspartyl residues in the template BNT1 (AHHAHHAADA) by the neutral Asparaginyl residues {BNTM, (AHHAHHAANA)} has no effect on either heme binding or β-hematin formation. Our findings suggest that weak dispersive forces like N-H···π and lp···π may assist in β-hematin formation. These findings may be helpful for rational drug design against malaria.

## Supporting Information

Figure S1 Characterization of BNT1 and BNTM by RPHPLC. Panels A and B show RPHPLC profiles of BNT1 and BNTM respectively. Analytical purity of Purified BNT1 and BNTM was assessed by chromatography on a reversed phase C_18_ column (Microsorb, 15×4.6 mm, 15 µ) using 0.1% TFA/water (solvent A) and 0.1% TFA/acetonitrile (solvent B) and running a 5–45% linear gradient over 20 minutes at a flow rate of 1 mL/min. Wavelength detector was set at 214 nm.
(TIF)

Figure S2 Mass Spectrum (ESI-MS) of peptide dendrimer BNT1 (panel A), BNTM (panel B) and BNTM (t) (panel C). (t) represents the sample of BNTM after it was treated under the acidic and high ionic strength conditions used to promote hemozoin formation (500 mM sodium acetate pH 5, 37°C, 24 hrs). Observed mass values for BNT1, BNTM and BNTM (t) were 4703 Da, 4698.43 Da and 4699.28 Da respectively. The difference of 4 Daltons between the observed masses of BNT1 and BNTM is as expected since replacement of four Aspartyl residues with four Asparginyl residues is consistent with a loss of four Daltons. The nearly identical masses of BNTM and BNTM(t) suggests that 500 mM sodium acetate pH 5, 37°C, 24 hrs- the conditions used to promote hemozoin formation did not cause deamidation of Asparginyl residues.
(TIF)

Figure S3 UV-Visible spectra of heme and heme-polyhistidine complex (A) and FTIR spectra (B) of polyhistidine mediated bicarbonate stable heme aggregates. Panel A shows heme-polyhistidine binding at pH 4.8 (500 mM acetate buffer). Bathochromic shifts from ∼384 to 415 nm indicate heme binding to polyhistidine. Panel B shows comparison of FTIR spectra of β-hematin (black line), polyhistidine meditaed heme aggregates (blue line) and heme (red line). Characteristic signatures peaks of β-hematin (1210 cm^−1^ and 1664 cm^−1^) are absent in both polyhistidine meditaed heme aggregates and heme.
(TIF)
