# Supplementary figures and images for: Dendrimeric Template of Plasmodium falciparum Histidine Rich Protein II Repeat Motifs Bearing Asp→Asn Mutation Exhibits Heme Binding and β-Hematin Formation
Source: PLoS One. 2014 Nov 14;9(11):e112087. doi: 10.1371/journal.pone.0112087 (PMC4232395; doi:10.1371/journal.pone.0112087)

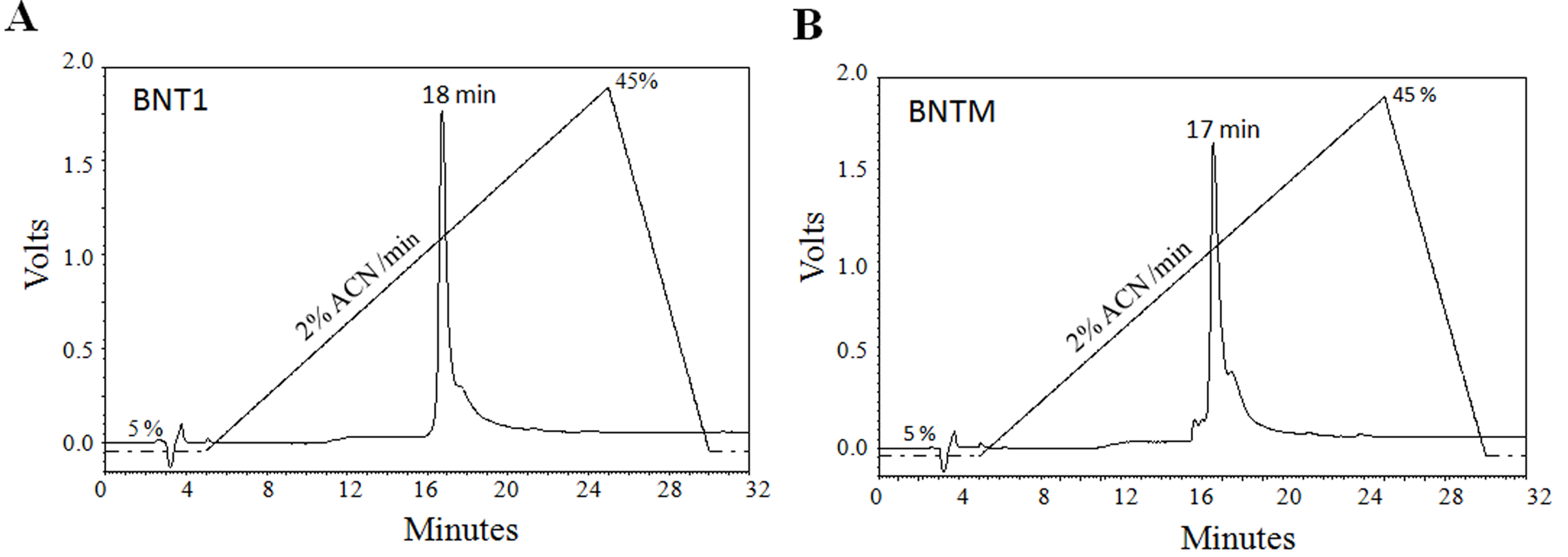

Supplement: Figure S1 — Characterization of BNT1 and BNTM by RPHPLC. Panels A and B show RPHPLC profiles of BNT1 and BNTM respectively. Analytical purity of Purified BNT1 and BNTM was assessed by chromatography on a reversed phase C18 column (Microsorb, 15×4.6 mm, 15 µ) using 0.1% TFA/water (solvent A) and 0.1% TFA/acetonitrile (solvent B) and running a 5–45% linear gradient over 20 minutes at a flow rate of 1 mL/min. Wavelength detector was set at 214 nm. (TIF) [file pone.0112087.s001.tif]

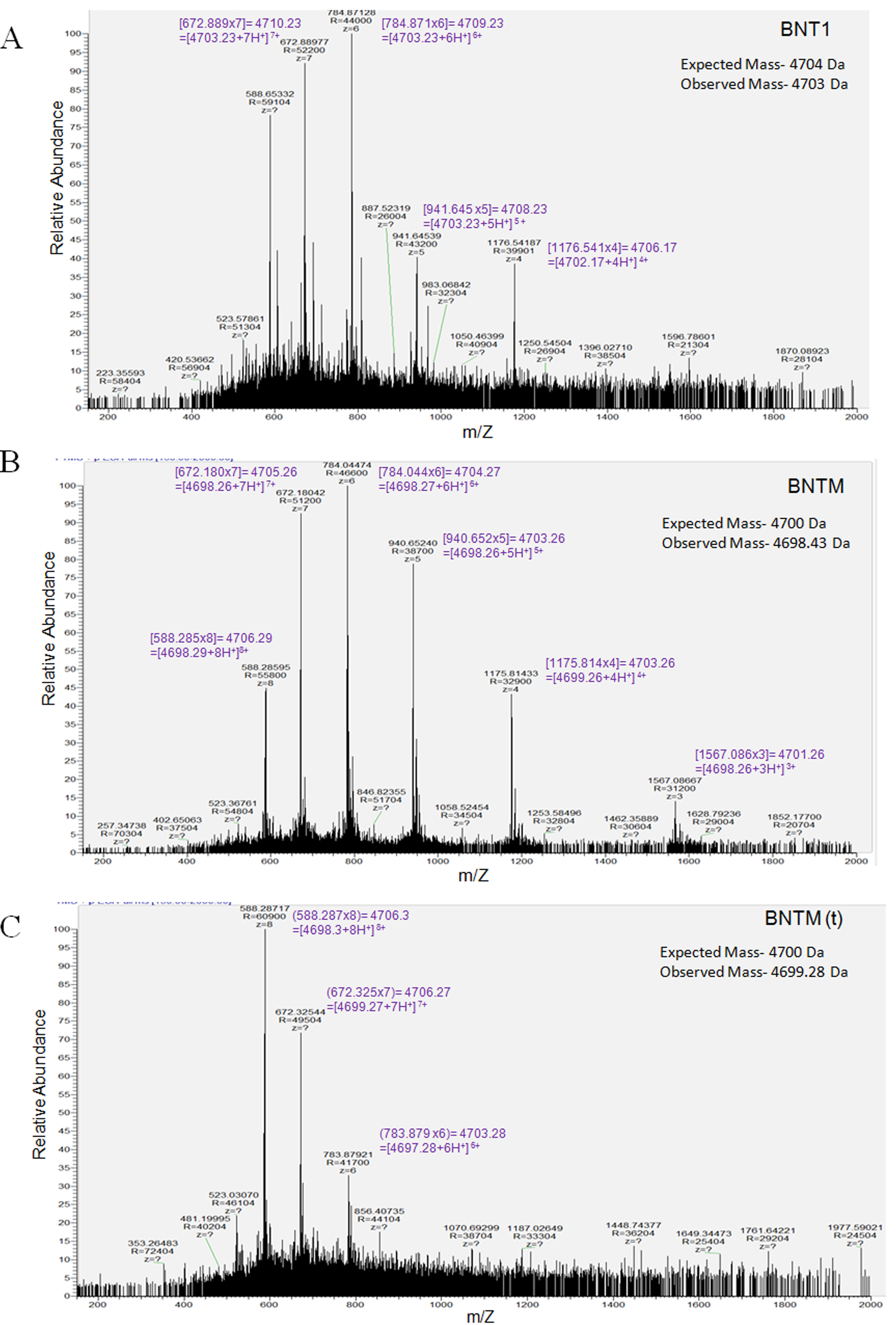

Supplement: Figure S2 — Mass Spectrum (ESI-MS) of peptide dendrimer BNT1 (panel A), BNTM (panel B) and BNTM (t) (panel C). (t) represents the sample of BNTM after it was treated under the acidic and high ionic strength conditions used to promote hemozoin formation (500 mM sodium acetate pH 5, 37°C, 24 hrs). Observed mass values for BNT1, BNTM and BNTM (t) were 4703 Da, 4698.43 Da and 4699.28 Da respectively. The difference of 4 Daltons between the observed masses of BNT1 and BNTM is as expected since replacement of four Aspartyl residues with four Asparginyl residues is consistent with a loss of four Daltons. The nearly identical masses of BNTM and BNTM(t) suggests that 500 mM sodium acetate pH 5, 37°C, 24 hrs- the conditions used to promote hemozoin formation did not cause deamidation of Asparginyl residues. (TIF) [file pone.0112087.s002.tif]

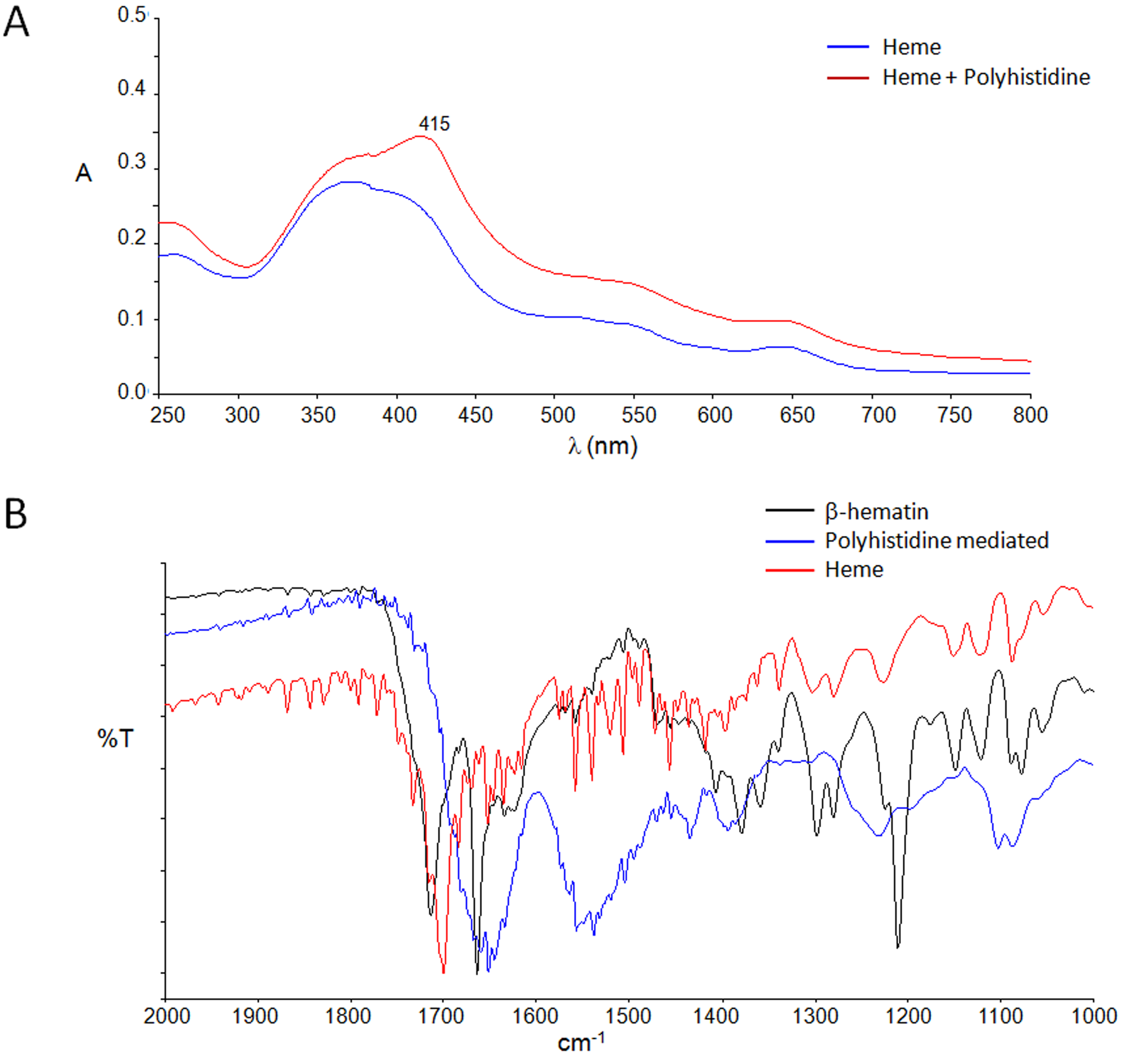

Supplement: Figure S3 — UV-Visible spectra of heme and heme-polyhistidine complex (A) and FTIR spectra (B) of polyhistidine mediated bicarbonate stable heme aggregates. Panel A shows heme-polyhistidine binding at pH 4.8 (500 mM acetate buffer). Bathochromic shifts from ∼384 to 415 nm indicate heme binding to polyhistidine. Panel B shows comparison of FTIR spectra of β-hematin (black line), polyhistidine meditaed heme aggregates (blue line) and heme (red line). Characteristic signatures peaks of β-hematin (1210 cm−1 and 1664 cm−1) are absent in both polyhistidine meditaed heme aggregates and heme. (TIF) [file pone.0112087.s003.tif]
